# The quintom theory of dark energy after DESI DR2

**DOI:** 10.1093/nsr/nwag115

**Published:** 2026-02-25

**Authors:** Yifu Cai, Xin Ren, Taotao Qiu, Mingzhe Li, Xinmin Zhang

**Affiliations:** Department of Astronomy, School of Physical Sciences, University of Science and Technology of China, Hefei 230026, China; CAS Key Laboratory for Research in Galaxies and Cosmology, School of Astronomy and Space Science, University of Science and Technology of China, Hefei 230026, China; Department of Astronomy, School of Physical Sciences, University of Science and Technology of China, Hefei 230026, China; CAS Key Laboratory for Research in Galaxies and Cosmology, School of Astronomy and Space Science, University of Science and Technology of China, Hefei 230026, China; Department of Astronomy, School of Physics, Huazhong University of Science and Technology, Wuhan 430074, China; Interdisciplinary Center for Theoretical Study & Peng Huanwu Center for Fundamental Theory, University of Science and Technology of China, Hefei 230026, China; Theoretical Physics Division, Institute of High Energy Physics, Chinese Academy of Sciences, Beijing 100049, China; School of Nuclear Science and Technology, University of Chinese Academy of Sciences, Beijing 101408, China

**Keywords:** quintom theory, dark energy, dark energy spectroscopic instrument

## Abstract

Observations from the Dark Energy Spectroscopic Instrument Data Release 2 (DESI DR2) challenge the $\Lambda$ cold dark matter ($\Lambda$CDM) paradigm by suggesting that the equation-of-state parameter of dark energy evolves across $w = -1$, a phenomenon known as the quintom scenario. Motivated by this development, we present a staged review of quintom cosmology, including its theoretical foundations, observational supports, implications and possible extensions. We first trace the historical progression from Einstein’s static cosmological constant to modern dynamical dark energy, summarizing recent cosmological constraints that favor an evolving $w(z)$ with time. A key focus is the theoretical no-go theorem for dark energy, which shows that no single canonical field or perfect-fluid model can smoothly cross the $w = -1$ boundary. We then survey viable quintom constructions, including two-field models, single-scalar-field models with higher derivatives, modified-gravity frameworks, interacting dark energy and effective field theory approaches that unify these mechanisms. Possible interactions between quintom fields and ordinary matter, as well as their potential roles in yielding non-singular universe solutions, are discussed.

## Teaser

Observations from DESI DR2 are challenging the ⋀CDM paradigm by suggesting that the equation-of-state parameter of dark energy evolves across w=-1, a phenomenon known as the Quintom scenario.

## INTRODUCTION

The accelerated expansion of the Universe was discovered in 1998 through measurements of distances from high-redshift Type Ia supernovae (SNe) [1,2], and was subsequently confirmed by observations of the cosmic microwave background (CMB) and other cosmological observations. The simplest explanation—a constant dark-energy $\Lambda$—was then introduced to describe this acceleration, forming the basis of the standard $\Lambda$ cold dark matter ($\Lambda$CDM) cosmological scenario.

The Dark Energy Spectroscopic Instrument (DESI) is a state-of-the-art astronomical instrument designed to carry out groundbreaking studies of the dark Universe. Installed on the Mayall telescope at Kitt Peak National Observatory, DESI’s primary mission is to create a detailed three-dimensional map of the universe by measuring the spectra of more than 30 million galaxies and quasars. By analyzing these spectra, scientists aim to shed light on the nature of dark energy, the mysterious force driving the accelerated expansion of the universe.

More recently, measurements of baryon acoustic oscillations (BAOs) from DESI 2024, when combined with SNe datasets, have provided evidence for dynamical dark energy at a confidence level of 2.5–$3.9\sigma$ [3]. In particular, the DESI 2024 data exhibit a preference for a quintom behavior [4], in which the equation-of-state (EoS) parameter of dark energy, *w*, crosses the cosmological-constant boundary ($w = -1$). This dynamical evolution of dark energy has attracted significant attention and stimulated extensive investigation.

The latest version of DESI Data Release 2, when analyzed together with SNe constraints, further reinforces this preference, increasing the statistical significance to more than $4\sigma$ [5–7]. These findings have motivated renewed interest in exploring models of dynamical dark energy capable of accommodating such behavior, highlighting the need for further theoretical studies of quintom dark energy [8–13].

This article is organized as follows. It first traces the development from Einstein’s cosmological constant to modern observational evidence for an evolving dark‐energy component, motivating a phenomenological classification of equations of state. It then confronts these possibilities with the latest high-precision datasets—DESI BAO, supernovae and the CMB—showing a growing preference for a quintom-B scenario. The no-go theorem that forbids $w=-1$ crossings in single‐field or single perfect‐fluid models is reviewed next, thereby setting the theoretical stage for viable quintom constructions. Concrete model‐building avenues are subsequently surveyed, including multi‐scalar systems, scalar fields with higher derivatives, modified-gravity realizations, interacting dark energy and an effective-field-theory unification. The implications of quintom cosmology are then considered from two perspectives. The first explores possible couplings between quintom dark energy to ordinary matter, outlining the resulting laboratory and cosmological signatures. The second discusses the role of quintom dynamics in non-singular early-universe scenarios, such as bouncing, cyclic and emergent solutions. The article concludes with a brief conclusion.

## FROM CONSTANT TO DYNAMICS

In 1917, Albert Einstein introduced a cosmological constant, $\Lambda$, in the development of general relativity. Before 1998, it was widely considered that cosmic expansion would slow due to gravitational attraction, and a deceleration parameter *q* was introduced to quantify this expected behavior. Surprisingly, cosmological measurements of distant supernovae revealed an unexpected negative value for *q*, indicating that the expansion of the Universe is not slowing down but instead accelerating. This discovery was later corroborated by independent probes, including observations of the CMB and large-scale structure (LSS), and naturally led to a profound conclusion: the Universe is currently undergoing accelerated expansion. To account for this phenomenon, the concept of dark energy was proposed, characterized as a smooth, spatially homogeneous energy component with negative pressure that dominates the present-day universe on large scales.

By introducing a constant term $\Lambda$ into general relativity to account for the observed acceleration of the Universe, the $\Lambda$CDM model provides a simple and empirically successful framework for modern cosmology. It assumes that general relativity is the correct theory of gravity on cosmological scales and that the Universe consists of radiation, ordinary matter, cold dark matter (CDM) and a cosmological constant $\Lambda$, which is associated with dark energy whose density remains constant even in an expanding background.

Within the framework of the $\Lambda$CDM cosmology, the evolution of the homogeneous and isotropic background universe is governed by the Friedmann equations


(1) \begin{eqnarray*}
H^2 &\equiv& \bigg (\frac{\dot{a}}{a}\bigg )^2=\frac{8\pi G\rho }{3},\\
\frac{\ddot{a}}{a} &=& -\frac{4\pi G}{3}(\rho +3p),
\end{eqnarray*}

where *H* is the Hubble parameter, and $\rho$ and *p* denote the total energy density and pressure of all components in the Universe at a given time. The acceleration of the universe requires $\ddot{a}>0$, which implies an effective EoS parameter $w = p/\rho < - 1/3$. The cosmological constant $\Lambda$ can therefore be interpreted as a dark-energy component with a constant equation of state $w_{\Lambda } = -1$.

Despite its remarkable success, the introduction of the cosmological constant $\Lambda$ presents profound theoretical challenges [14]. One is the well-known cosmological-constant problem: quantum mechanically, field theories generically predict a vacuum energy density more than 120 orders of magnitude larger than the observed value, $\rho _{\Lambda }^{\mathrm{obs}} \sim (10^{-3}\ \mathrm{eV})^{4}$. This framework also suffers from the cosmic coincidence problem, namely why the energy density of dark energy is of the same order of magnitude as that of matter precisely at the present epoch.

Except for the aforementioned conceptual issues, several observational tensions or anomalies have emerged, particularly in recent years [15,16], including the Hubble tension, the $\sigma _8$ tension, large-angle anomalies in the CMB and a possible cosmic dipole. These discrepancies suggest that the prevailing $\Lambda$CDM model may be incomplete and motivate the exploration of its extensions. In the literature, numerous studies have been devoted to addressing some of these challenges, among which dynamic models of dark energy—whose *w* is not always equal to $-1$—have attracted considerable attention [17].

In general, dark-energy models can be phenomenologically categorized into the following main classes:

-
$w = -1$ : corresponding to the cosmological constant $\Lambda$;
-
$w > -1$ : the EoS lies above the cosmological-constant boundary, usually referred to as quintessence dark energy;
-
$w < -1$ : the EoS lies below the cosmological-constant boundary, usually referred to as phantom dark energy;
- *w* crosses $-1$: the EoS evolves across the cosmological-constant boundary, usually referred to as quintom dark energy. If the crossing occurs from above to below with time, it is known as quintom-A; if the crossing occurs from below to above, it is known as quintom-B.

Different values of *w* also introduce distinct theoretical challenges. For example, when $w < -1$, the null energy condition (NEC) is commonly violated [18], making phantom energy difficult to realize using ordinary matter fields, although such violations may be avoided under certain circumstances [19].

Given the absence of a fundamental theory that naturally explains the origin of dark energy, phenomenologists often adopt a pragmatic approach by constructing models based on its macroscopic behavior and constraining the relevant parameters using observational data. A common example is the *w*CDM model, which assumes a constant but arbitrary EoS $w\ne -1$. A more general class is the $w_0w_a$CDM model, in which the evolution of *w* with time is described by two parameters. One of the most widely used parameterizations for dynamical dark energy is the Chevallier–Polarski–Linder (CPL) parameterization [20,21], which expresses $w(z)$ as


(2) \begin{eqnarray*}
w(z)=w_0+w_a(1-a).
\end{eqnarray*}

With the advent of precision cosmology, between 2003 and 2012 WMAP provided increasingly stringent constraints on both the *w*CDM and $w_0w_a$CDM models. Nevertheless, these analyses did not reveal significant deviations from the $\Lambda$CDM model, as summarized in Table 1. When deriving cosmological parameter constraints, WMAP results were frequently combined with additional datasets, including BAO measurements from surveys such as 2dFGRS and 6dFGS, as well as SN datasets such as SNLS and Union2, together with small-scale CMB data and independent measurements of the Hubble constant $H_0$.

**Table 1.** Developments in observation constraints on the dark-energy EoS from WMAP 2003–12.

Data	*w*	$w_0$	$w_a$
WMAP1 [22]	$-0.98\pm 0.12$	-	-
WMAP3 [23]	$-0.967{}^{+0.073}_{-0.072}$	-	-
WMAP5 [24]	$-1^{+0.12}_{-0.14}$	$-1.06\pm 0.14$	$0.36\pm 0.62$
WMAP7 [25]	$-1.10\pm 0.14$	$-0.93\pm 0.13$	$w = -\, 0.41^{+\, 0.72}_{-\, 0.71}$
WMAP9 [26]	$-1.073^{+0.090}_{-0.089}$	-	-

In 2013, Planck released its first-year data [27], yielding $w= -1.13^{+0.13}_{-0.14}$ (Planck2013 + WMAP9 + SNLS), which lies within $2\sigma$ of the phantom regime. Subsequent improvements in supernova calibration led to the Joint Light-curve Analysis (JLA) dataset released in 2014 [28], which significantly improved the spectral calibration of SNLS data and exhibited a $1.8\sigma$ discrepancy relative to SNLS-3. Incorporating JLA data into the Planck2013 analysis brought the constraints on dark energy back into agreement with $\Lambda$CDM. In Planck2015 [29,30], which adopted JLA as its default SN dataset, the previous SNLS-induced deviation from the standard model was similarly resolved, yielding a final result constraint of $w = -1.006^{+0.085}_{-0.091}$.

Moreover, the emergence of new observational datasets, including DESY1, Pantheon, Planck2018 and DESY3, further constrained the *w*CDM and $w_0w_a$CDM models. Across these studies, the joint analyses continued to show no significant deviation of *w* from $-1$. In 2022, the Pantheon+ supernova compilation [31] reported $w= -0.90\pm 0.14$ (SN only) and $w= -0.978^{+0.024}_{-0.031}$ when combined with CMB and BAO data. Although the fitted values of $w_0$ and $w_a$ remained consistent with $\Lambda$CDM within the $2\sigma$ confidence level, the results exhibited a mild deviation relative to previous datasets. Meanwhile, this trend was further supported by the Union3 compilation [32], which yielded constraints on the $w_0w_a$CDM model showing a mild tension with $\Lambda$CDM at the 1.7–$2.6\sigma$ level, favoring models with $w_0> -1$ and $w_a< 0$. Taken together, these results point to an evolving dark-energy component whose EoS increases with time, suggesting a present-day value $w> -1$.

In 2024, following the indications of dynamical dark energy from the Pantheon+ and Union3 compilations, DESY5 analyses showed that, whether using supernova data alone or in combination with CMB, BAO and $3\times 2pt$ measurements, the best-fit values of *w* are consistently slightly greater than $-1$ at more than the $1\sigma$ level. These findings are consistent with those obtained from the Union3 compilation, further supporting a mildly dynamical dark-energy scenario. In the same year, DESI released its first-year BAO measurements [3], which yielded constraints on $w_0$ and $w_a$ deviating from the standard model at the $2.6\sigma$, $2.5\sigma$, $3.5\sigma$ and $3.9\sigma$ levels when combined with CMB, Pantheon+, Union3 and DESY5 data, respectively. These results favor a dynamical dark-energy scenario characterized by $w_0 > -1, w_a < 0, w_0+w_a< -1$, i.e. a quintom-B scenario.

The DESI DR2 baryon acoustic oscillation measurements [5], when combined with CMB data alone, prefer $w_0>-1$ and $w_a<0$ over the $\Lambda$CDM model at a significance level of $3.1\sigma$ within the CPL parameterization, suggesting an evolving dark-energy component. When combined with Pantheon+, Union3 and DESY5, the significance of the preference for a dynamical dark-energy model reaches $2.8\sigma$, $3.8\sigma$ and $4.2\sigma$ within the CPL parameterization, respectively. Compared with DR1, DR2 exhibits improved precision and reduced uncertainties. Following the release of DESI BAO DR2, an extended analysis [6] further investigated the behavior of dark energy and confirmed the evidence for dynamical dark energy.

Researchers are eager to uncover the potential new physics underlying these discrepancies and to explore their fundamental nature. To realize the dynamical behavior of dark energy, a variety of theoretical models have been proposed. Representative scalar-field models include quintessence [33,34], phantom [35], quintom [4,36] and k-essence [37,38]. In addition, some approaches attribute the driving force of cosmic acceleration to modifications of general relativity, attempting to reproduce the accelerated expansion through the geometric structure of gravity.

## QUINTOM DARK ENERGY UNDER THE LATEST OBSERVATIONS

Last year, BAO measurements from DESI suggested dynamical dark energy at the 2.5–$3.9\sigma$ level when combined with SNe datasets [3], generating considerable attention and discussion. The latest DESI Data Release 2, when combined with supernova constraints, strengthens this preference to up to $4.2\sigma$ [5–7], thereby motivating further investigations into dynamical dark energy. Subsequent work has carried out comprehensive observational analyses using a variety of cosmological datasets and phenomenological parameterizations. Intriguingly, the DESI data favor a quintom behavior [4], in which the dark-energy EoS parameter crosses the cosmological-constant boundary $w = -1$ from below. Meanwhile, a wide range of theoretical models beyond the cosmological constant have been explored in conjunction with DESI observations to explain this dynamical evolution of dark energy, including various extended scalar-field models, modified gravity and interacting dark-energy scenarios.

**Figure 1.**
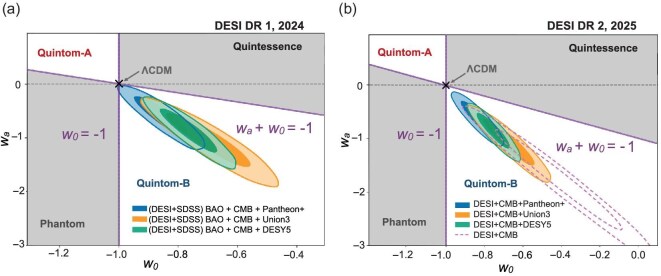
The 68% and 95% marginalized posterior constraints in the $w_0$-$w_a$ plane for the flat $w_0w_a$ model, obtained from the combination of DESI BAO data, CMB and SNe, are shown for Pantheon+ [31], Union3 [32] and DESY5 [39] SNe datasets. Panel (a) is taken from Adame *et al.* [3], while panel (b) is from Abdul Karim *et al.* [5]. Boundary lines have been added to illustrate the regions corresponding to quintessence, phantom and quintom dark energy.

We present an overview of the cosmological constraints on the $w_0w_a$ dark-energy model based on DESI observations. The constraints derived from the two DESI data releases are summarized in Fig. 1. For clarity, dividing lines have been added to partition the parameter space into four distinct regions, corresponding to quintessence, phantom, quintom-A and quintom-B; the origin denotes the $\Lambda$CDM model. As shown in Fig. 1, the $\Lambda$CDM model lies well outside the $2\sigma$ credible region allowed by the posterior constraints, with both data releases favoring the quintom-B regime.

The CPL parametrization fixes the higher-order terms in the Taylor expansion. As such, it serves as an effective low-redshift parameterization and does not introduce additional information from higher order terms. To examine the possible higher-order effects and avoid such a bias, one may extend the parametrization to include higher-order terms or instead adopt data-driven approaches [41].

**Figure 2.**
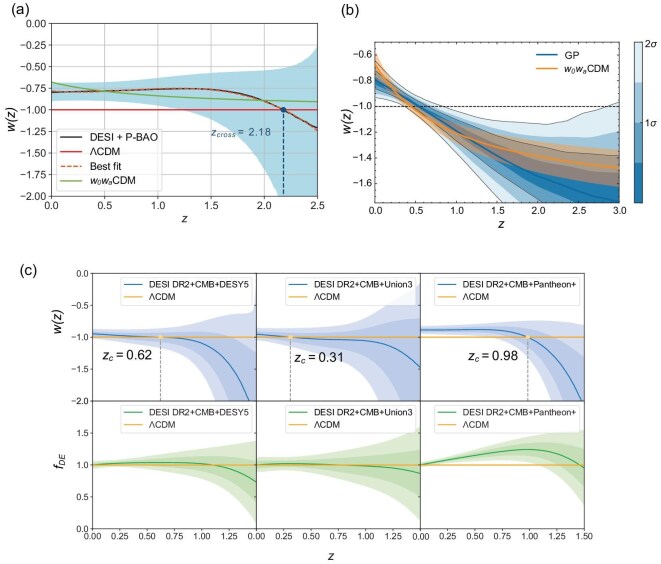
The reconstructed EoS parameter *w* obtained from Gaussian process regression. Panel (a) is from Fig. 2 of [11], where the curve denotes the mean value and the shaded region indicates the allowed range at the 68% confidence interval. Panel (b) is from Fig. 10 of [6], in which the Gaussian process reconstruction is shown accompanied by shaded 68% and 95% confidence intervals. Panel (c) is from Fig. 3 of [40] and shows the mean values of the reconstructed dark-energy EoS parameter *w* and the normalized energy density $f_{de}$, together with the $1\sigma$ and $2\sigma$ uncertainties, where $f_{de}(z)=\rho _{de}(z)/\rho _{de,0}$.

Consequently, non-parametric approaches are both important and necessary. One widely used non-parametric method is Gaussian process regression [42,43], which allows the reconstruction of a function and its derivatives in a model-independent manner from observational data, given a chosen kernel covariance function. The results obtained from Gaussian process regression are shown in Fig. 2. The results from this non-parametric approach are consistent with those derived from the $w_0w_a$ parameterization.

**Figure 3.**
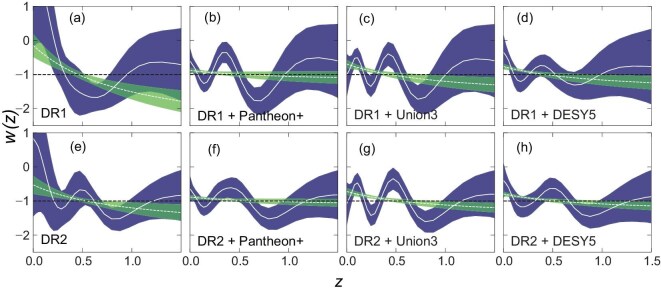
Dark-energy equation of state $w(z)$ reconstructed from several datasets. The results are shown for two different approaches: the correlation-prior method (lower, dark-blue band) and the ($w_0$, $w_a$) parameterization (upper, green band). The panel is taken from Fig. 3 of [7].

In [7], the DESI collaboration performed a non-parametric Bayesian reconstruction of $w(z)$ using principal component analysis [44], based on a joint analysis of DESI BAO, SNe and CMB data, as shown in Fig. 3. The reconstructed $w(z)$CDM models yield results consistent with those of the companion DESI papers. The statistical significance of deviations from $w\ne -1$ reaches $4.3 \sigma$ for DESI DR2 BAO + DESY5, $3.9 \sigma$ for DESI DR2 BAO + Union3 and $3.1 \sigma$ for DESI DR2 BAO + Pantheon+, favoring dynamical dark energy with an EoS parameter crossing $-1$. Using the same methodology, this preference for dynamical dark energy was reported in 2012, with a significance of $2.5 \sigma$ from combined SNLS3 data with weak prior [45], and in 2017 with a significance of $3.5 \sigma$ from the combined ALL16 dataset [46]. Different parameterizations and non-parametric reconstructions, using various data combinations including DESI data, consistently yield a quintom dark-energy evolutionary behavior, demonstrating the robustness of this feature [7,47].

## QUINTOM MODELS AND THE NO-GO THEOREM

To examine the physical implications of quintom dark energy, it is essential to revisit the no-go theorem associated with dynamical dark energy. As reviewed in [48] (see also [49]), basic single-field models or single perfect-fluid models cannot exhibit quintom behavior because the no-go theorem strictly forbids the EoS parameter *w* from crossing the cosmological-constant boundary in such simple frameworks [50]. In general, dark energy is treated as an additional non-interacting fluid (alongside matter and radiation). In the conformal Newtonian gauge, ${\rm d}s^2=-a(\eta )^2 [ -(1+2\Psi ) {\rm d} \eta ^2+(1-2\Phi )\delta _{ij} {\rm d} x^i {\rm d} x^j ]$, and in the absence of anisotropic perturbations (so that $\Psi =\Phi$), dark-energy perturbations in Fourier space can be described as [51]


(3) \begin{eqnarray*}
\delta ^{\prime } = - \bigg (1+\frac{\bar{p}}{\bar{\rho }}\bigg )(\theta -3\Phi ^{\prime })-3\mathcal {H} \bigg (\frac{\delta p}{ \delta \rho }-\frac{\bar{p}}{\bar{\rho }} \bigg )\delta ,\\
\end{eqnarray*}

(4) \begin{eqnarray*}
\theta ^{\prime }= -\bigg (\mathcal {H}+\frac{\bar{p}^{\prime }}{\bar{\rho }+\bar{p}}\bigg )\theta + k^2 \bigg ( \frac{\delta p}{\bar{\rho }+\bar{p}} + \Psi \bigg ),\\
\end{eqnarray*}

where a prime denotes differentiation with respect to conformal time $\eta$, satisfying $ad\eta =dt$; $\mathcal {H}$ is the conformal Hubble parameter and $\delta \equiv \delta \rho / \bar{\rho }$, $\theta \equiv ik^{j}\delta T^{0}_{j}/(\bar{\rho }+\bar{p})$ denote the density contrast and velocity perturbation, respectively.

One may consider a barotropic perfect fluid characterized by its pressure $\bar{p}$, energy density $\bar{\rho }$ and EoS parameter $w=\bar{p}/\bar{\rho }$. The adiabatic sound speed for such a fluid is given by


(5) \begin{eqnarray*}
c_a^2=\frac{\delta p}{\delta \rho }\bigg |_{\rm {adiabatic}}=\frac{\bar{p}^{\prime }}{\bar{\rho }^{\prime }}=w-\frac{w^{\prime }}{3\mathcal {H}(1+w)}.
\end{eqnarray*}

It should be noted that, when *w* crosses $-1$, this expression for the sound speed diverges, leading to unphysical instabilities in dark-energy perturbations.

If the fluid is non-barotropic, dark-energy perturbations will naturally induce entropy variations. In such a case, dark-energy perturbations generate non-adiabatic (isocurvature) modes. A more general definition of the sound speed follows from the relation between the gauge-invariant pressure $\delta \hat{p}$ and energy density $\delta \hat{\rho }$,


(6) \begin{eqnarray*}
\delta \hat{p} = \delta p+3\mathcal {H}c_a^2(1+w)\bar{\rho }\frac{\theta }{k^2},
\end{eqnarray*}

(7) \begin{eqnarray*}
\delta \hat{\rho } = \delta \rho +3\mathcal {H}(1+w) \bar{\rho }\frac{\theta }{k^2}.
\end{eqnarray*}

In the rest frame, the gauge-invariant terms $\delta \hat{p}$ and $\delta \hat{\rho }$ coincide with the pressure *p* and energy density $\rho$ of the fluid; thus we define


(8) \begin{eqnarray*}
c_s^2=\frac{\delta \hat{p}}{\delta \hat{\rho }}=\frac{\delta p}{\delta \rho } \bigg |_{\rm {rest-frame}}.
\end{eqnarray*}

Consequently, the relation between $\delta p$ and $\delta \rho$ in a general frame can be written as


(9) \begin{eqnarray*}
\delta p = c_s^2 \delta \rho +\left(c_s^2-c_a^2 \right)[ 3 \mathcal {H}(1+w) \bar{\rho }]\frac{\theta }{k^2}.
\end{eqnarray*}

Substituting this relation into Equation (3), we obtain


(10) \begin{eqnarray*}
\theta ^{\prime }&=&-\bigg [\mathcal {H}-3\mathcal {H}(c_s^2-c_a^2+w)+\frac{w^{\prime }}{1+w} \bigg ]\theta \\
&& +\, k^2 \bigg (\frac{c_s^2}{1+w}\delta + \Psi \bigg ) \\
&=& -\mathcal {H}(1-3w)\theta +k^2 \Psi \\
&& +\, \frac{1}{1+w}\Big[3\mathcal {H}(1+w) \left(c_s^2-c_a^2 \right)\theta -w^{\prime }\theta\\
&& +\, k^2 c_s^2\delta \Big] \\
&=& -\mathcal {H}\theta +k^2\Psi +\frac{k^2\delta \hat{p}}{(1+w)\bar{\rho }}.
\end{eqnarray*}

From the definition of the velocity perturbations and Equation (9), it follows that $\theta$ and $\theta ^{\prime }$ diverge when crossing the cosmological-constant boundary, unless $\delta \hat{p}=0$ at the crossing point. The gauge-invariant entropy perturbation $\hat{\Gamma }$ is defined as


(11) \begin{eqnarray*}
\hat{\Gamma }=\frac{1}{w\bar{\rho }} \left(\delta p -c_a^2 \delta \rho \right)=\frac{1}{w \bar{\rho }} \left(\delta \hat{p}-c_a^2\delta \hat{\rho } \right).
\end{eqnarray*}

Because the adiabatic sound speed $c_a^2$ diverges at the crossing point, obtaining a finite value of $\hat{\Gamma }$ requires $\delta \hat{\rho }=0$. However, under this condition one necessarily has $\delta p = c_a^2\delta \rho$, which contradicts the assumption of non-adiabatic perturbations. Therefore, a smooth crossing with non-adiabatic perturbations is not allowed. In summary, for a single perfect fluid, it is impossible to realize a crossing of $w=-1$.

An analogous divergence arises for a generic single scalar field without higher-derivative terms. In general, the no-go theorem states that, in the Friedmann–Robertson–Walker (FRW) Universe described by a single perfect fluid or a single scalar field $\phi$ with Lagrangian $L = L(\phi , \partial _\mu \phi \partial ^\mu \phi )$, minimally coupled to Einstein gravity, the equation of state *w* cannot cross the cosmological-constant boundary [48,49].

## MODEL BUILDING OF QUINTOM DARK ENERGY

This no-go theorem shows explicitly that, to realize the quintom scenario, additional degrees of freedom must be introduced—either through higher-derivative terms in the matter sector, through multiple fields to ensure a healthy dispersion relation when crossing the cosmological boundary or via modified gravity, in which dark energy is treated as an effective description of physics beyond general relativity. In the following, we several several examples of quintom dark energy.

### Multi-field model

The first quintom dark-energy model was proposed in April 2004 [4]. It combines a quintessence field $\phi$ with a phantom field $\sigma$:


(12) \begin{eqnarray*}
S &=& \int d^4 x \sqrt{-g}\, \bigg [-\frac{1}{2} \nabla _\mu \phi \nabla ^\mu \phi\\
&&+\, \frac{1}{2} \nabla _\mu \sigma \nabla ^\mu \sigma -V(\phi , \sigma )\bigg ].
\end{eqnarray*}

The corresponding effective energy density $\rho$ and effective pressure *p* are given by


(13) \begin{eqnarray*}
\rho &=& \frac{1}{2} \dot{\phi }^2-\frac{1}{2} \dot{\sigma }^2+V(\phi , \sigma ),\\
\\
p &=& \frac{1}{2} \dot{\phi }^2-\frac{1}{2} \dot{\sigma }^2-V(\phi , \sigma ),
\end{eqnarray*}

and the associated EoS is


(14) \begin{eqnarray*}
w = \frac{p}{\rho }=\frac{\dot{\phi }^2- \dot{\sigma }^2-2 V(\phi , \sigma )}{\dot{\phi }^2-\dot{\sigma }^2+2 V(\phi , \sigma )}.
\end{eqnarray*}

There are also other variations of the double-field quintom model [36,52]. In addition, a generalized multiple-scalar-field model $\phi _i$ can be written as


(15) \begin{eqnarray*}
S &=& \int d^4 x \sqrt{-g}\,\bigg [-\frac{1}{2} \sum _i \epsilon _i \nabla _\mu \phi _i \nabla ^\mu \phi _i\\
&&- \, V({\phi _1,\phi _2,\dots ,\phi _n}) \bigg ],
\end{eqnarray*}

where


\begin{eqnarray*}
\epsilon _i= \left\lbrace \begin{array}{@{}l@{\quad }l@{}}+1& \mathrm{quintessence}, \\
-1& \mathrm{phantom} \end{array}\right.
\end{eqnarray*}

distinguishes between quintessence and phantom fields. The associating quantities are given by


(16) \begin{eqnarray*}
\rho _{\rm total} = \frac{1}{2} \sum _i \epsilon _i \dot{\phi }_i^2+V({\phi _1,\phi _2,\dots ,\phi _n}),
\end{eqnarray*}

(17) \begin{eqnarray*}
p_{\rm total} = \frac{1}{2} \sum _i \epsilon _i \dot{\phi }_i^2-V({\phi _1,\phi _2,\dots ,\phi _n}),
\end{eqnarray*}

(18) \begin{eqnarray*}
w_{\rm total} = \frac{\sum _i \epsilon _i \dot{\phi }_i^2-2 V({\phi _1,\phi _2,\dots ,\phi _n})}{\sum _i \epsilon _i \dot{\phi }_i^2+2 V({\phi _1,\phi _2,\dots ,\phi _n})}.
\end{eqnarray*}

### Single scalar field with higher-derivative terms

A single-scalar-field quintom model with higher-derivative terms was proposed in [53]. In DHOST [54], Horndeski [55] and Galileon [56] theories, the Lagrangian is carefully constructed to include couplings between the kinetic term, $X \equiv \partial _\mu \phi \partial ^\mu \phi /2$, and the higher-derivative term $\Box \phi$, allowing the EoS to cross $-1$ without introducing pathologies such as ghost instabilities.

Specifically, we consider the model with Lagrangian [57]


(19) \begin{eqnarray*}
L= -X+c_1X\Box \phi +c_2X\phi ^2,
\end{eqnarray*}

where $c_{1,2}$ are constants. From the Lagrangian, the pressure and energy density are obtained as


(20) \begin{eqnarray*}
\rho =&(c_2\phi ^2-1+6c_1H\dot{\phi })X,
\end{eqnarray*}

(21) \begin{eqnarray*}
p=&(c_2\phi ^2-1-2c_1\ddot{\phi })X.
\end{eqnarray*}

Thus, the EoS is


(22) \begin{eqnarray*}
w\equiv \frac{p}{\rho }=\frac{c_2\phi ^2-1-2c_1\ddot{\phi }}{c_2\phi ^2-1+6c_1H\dot{\phi }},
\end{eqnarray*}

where an overdot denotes differentiation with respect to cosmic time *t*. However, for comparison with the data, it is convenient to transfer the variable from *t* to the scale factor *a*. Accordingly, Equation (21) becomes


(23) \begin{eqnarray*}
w&\equiv& \frac{p}{\rho } \\
&=&\frac{c_2\phi ^2-1-c_1(2aH^2\phi ^{\prime }+a^2(H^2)^{\prime }\phi +2a^2H^2\phi ^{\prime \prime })}{c_2\phi ^2-1+6c_1aH^2\phi ^{\prime }},\\
\end{eqnarray*}

where a prime denotes differentiation with respect to *a*.

The dynamics of this model were analyzed in [57]. One may set the initial condition of $\phi$ to be $\phi _i=0$ at $a_i$. To ensure the positivity of the energy density, the condition $6c_1aH^2\phi ^{\prime }>1$ must be satisfied. Moreover, following the analysis in [57], the velocity of $\phi$ can be set as $\phi ^{\prime }=A/aH^2$. Therefore during the matter-dominated era, where $(H^2)^{\prime }=-3H^2/a$, one obtains


(24) \begin{eqnarray*}
w\simeq \frac{-1-3c_1A}{-1+6c_1A}.
\end{eqnarray*}

In the limit $c_1A\rightarrow 1/3$, one finds that $w\rightarrow -2$ [57]. On the other hand, a positive $\phi ^{\prime }$ causes $\phi$ to increase, and when the $c_2\phi ^2$ term in both *p* and $\rho$ dominates over the remaining terms, the equation of state *w* will reach 1. Consequently, a crossing of $w=-1$ arises naturally in this model.

To compare with recent DESI data, we expand the equation of state around $a=1$. From Equation (22), with $\phi ^{\prime }=A/aH^2$, one obtains


(25) \begin{eqnarray*}
\phi (a)&=&\int \frac{Ada}{aH^2} \\
&\simeq& \frac{Ae^{6w_{T0}}}{2}(a-a_i)[2(a+a_i-1)\\
&&+\, 3w_{T0}(a+a_i-2)],
\end{eqnarray*}

where $w_{T0}$ denotes the total equation of state of the Universe today. Then *w* can be expressed as a function of $A,c_1,c_2$ and $a_i$. Compared with the CPL parametrization, $w=w_0+w_a(1-a)$, the corresponding expressions for $w_0$ and $w_a$ can be derived. Consequently, one can invert these relations to solve for the coefficients $c_{1,2}$, expressing them in terms of $w_0$ and $w_a$.

**Figure 4.**
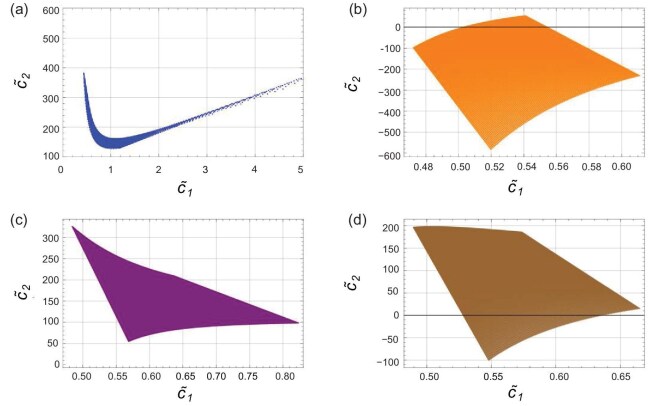
Constraints on the coefficients $\tilde{c}_1$ and $\tilde{c}_2$ from DESI+CMB data (a), DESI+CMB+Pantheon+ data (b), DESI+CMB+Union3 data (c) and DESI+CMB+DESY5 data (d), respectively. We choose $a_i=0.5$, corresponding to redshift $z_i\simeq 1$, which lies in the matter-dominated era. The total equation of state $w_{T0}\simeq w_0\Omega _{DE0}$, where $\Omega _0$ denotes the present dark-energy density fraction, $\Omega _{DE0}\simeq 0.647$.

The DESI data provide the following constraints on $w_0$ and $w_a$: $w_0=-0.42\pm 0.21$, $w_a= -1.75 \pm 0.58$ (DESI+CMB); $w_0=-0.838 \pm 0.055$, $w_a=-0.62^{+0.22}_{-0.19}$ (DESI+CMB+Pantheon+); $w_0=-0.667\pm 0.088$, $w_a=-1.09^{+0.31}_{-0.27}$ (DESI+CMB+Union3); $w_0=-0.752\pm 0.057$, $w_a=-0.86^{+0.23}_{-0.20}$ (DESI+CMB+DESY5) [5]. Using these observational constraints, we further restrict the model parameters $c_{1,2}$. The numerical constraints are shown in Fig. 4. Note that $c_{1,2}$ are degenerate with the overall factor of the $\phi$ field, namely *A*; thus, we effectively constrain the combined parameters $\tilde{c}_1\equiv c_1A$ and $\tilde{c}_2\equiv c_2A^2$.

Figure 4 shows the parametric region of $\lbrace \tilde{c}_1,\tilde{c}_2\rbrace$ constrained by DESI+CMB, DESI+CMB+Pantheon+, DESI+CMB+Union3 and DESI+CMB+DESY5 data. One sees that a large parameter space in this model remains compatible with the current DESI DR2 data. Moreover, the perturbations of this model can be examined to verify the absence of pathological instabilities. The perturbed Lagrangian derived from Equation (18) is given by [57]


(26) \begin{eqnarray*}
\delta L\sim D [(\partial _t{\delta \phi })^2-a^{-2}c_s^2(\partial _i\delta \phi )^2],
\end{eqnarray*}

where $\delta \phi$ denotes the perturbation of the $\phi$ field, and


(27) \begin{eqnarray*}
D = c_2\phi ^2-1+6c_1H\dot{\phi },
\end{eqnarray*}

(28) \begin{eqnarray*}
c_s^2 = \frac{c_2\phi ^2-1+2c_1(\ddot{\phi }+2H\dot{\phi })}{c_2\phi ^2-1+6c_1H\dot{\phi }}.
\end{eqnarray*}

It is evident that if $D>0$, no ghost instability arises, and if $c_s^2>0$, no gradient instability occurs.

**Figure 5.**
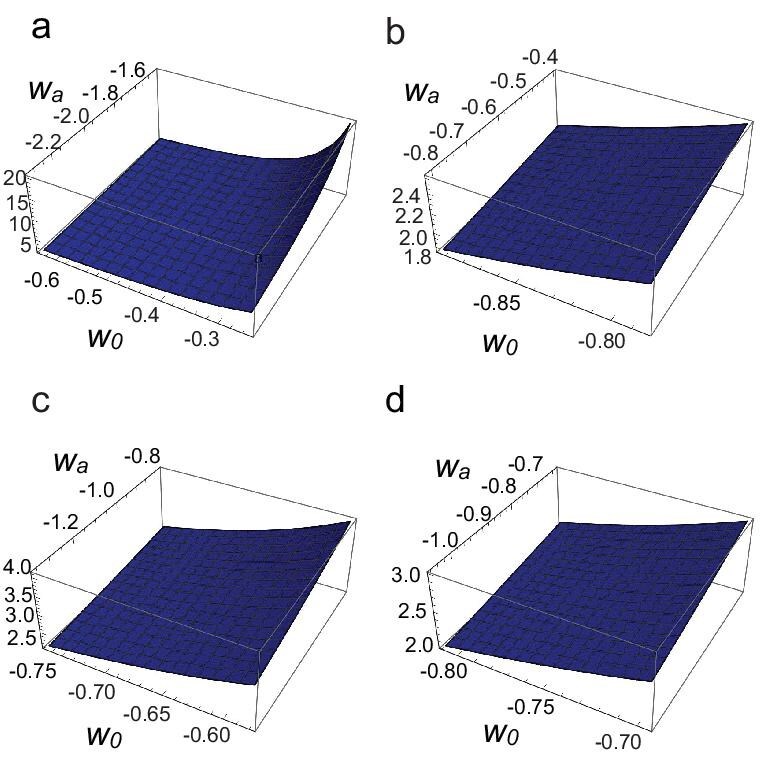
The value of the factor *D* (vertical axis) as a function of $w_0$ and $w_a$ within the allowed parameter space of DESI+CMB data (a), DESI+CMB+Pantheon+ data (b), DESI+CMB+Union3 data (c) and DESI+CMB+DESY5 data (d). The parameter choices are the same as those in Fig. 4.

**Figure 6.**
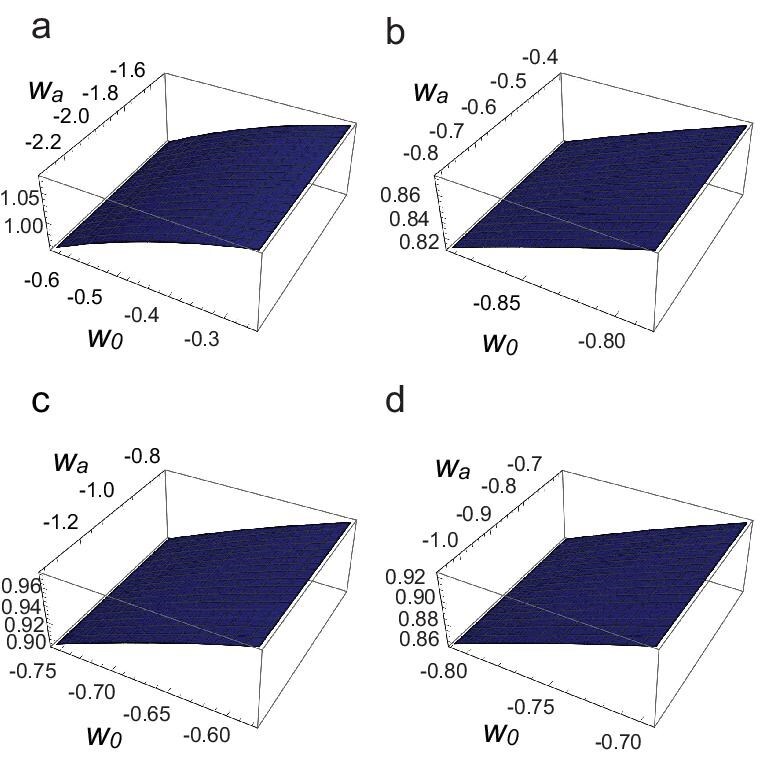
The value of the factor $c_s^2$ (vertical axis) as a function of $w_0$ and $w_a$ within the allowed parameter space of DESI+CMB data (a), DESI+CMB+Pantheon+ data (b), DESI+CMB+Union3 data (c) and DESI+CMB+DESY5 data (d). The parameter choices are the same as those in Fig. 4.

Although it is difficult to analyse the positivity conditions analytically, by adopting the ansatz $\phi ^{\prime }=A/aH^2$ and expressing the result in terms of $c_{1,2}$, we can express *D* and $c_s^2$ in terms of $w_0$ and $w_a$. We then plot these quantities as functions of $w_0$ and $w_a$ within the DESI DR2 allowed region, as shown in Figs 5 and 6. From the figures we see that throughout the allowed regions one always has $D>0$ and $c_s^2>0$, demonstrating that the model is free from instabilities.

### Modified-gravity models

Besides matter-field constructions, the modified gravity (MG) provides another approach to generating dark-energy effects and thus the quintom scenario, giving dark energy a gravitational interpretation. A commonly considered framework is metric-affine gravity (MAG) [58], in which gravity is described by a metric and a general affine connection. In this formalism, the general affine connection $\Gamma ^{\alpha }_{\mu \nu }$ can be decomposed as


(29) \begin{eqnarray*}
\Gamma ^{\alpha }_{\mu \nu } = \mathring{\Gamma }^{\alpha }_{\mu \nu } +L^{\alpha }_{\mu \nu }+K^{\alpha }_{\mu \nu },
\end{eqnarray*}

where $\mathring{\Gamma }^{\alpha }_{\mu \nu }$ is the Levi–Civita connection, and $L ^{\alpha }_{\mu \nu }$ and $K^{\alpha }_{\mu \nu }$ are the disformation tensor and contortion tensors, respectively, characterizing the deviation of the full affine connection from the Levi–Civita connection. The general affine connection defines the curvature, torsion and non-metricity tensors as [59]


(30) \begin{eqnarray*}
R^{\sigma }_{\ \ \rho \mu \nu } &\equiv& \partial _{\mu } \Gamma ^{\sigma }_{\ \ \nu \rho } - \partial _{\nu } \Gamma ^{\sigma }_{\ \ \mu \rho } + \Gamma ^{\alpha }_{\ \ \nu \rho } \Gamma ^{\sigma }_{\ \ \mu \alpha } \\
&&-\, \Gamma ^{\alpha }_{\ \ \mu \rho } \Gamma ^{\sigma }_{\ \ \nu \alpha },
T^{\lambda }_{\ \ \mu \nu } \equiv \Gamma ^{\lambda }_{\ \ \nu \mu }-\Gamma ^{\lambda }_{\ \ \mu \nu }, \\
Q_{\rho \mu \nu } &\equiv& \nabla _{\rho } g_{\mu \nu } = \partial _\rho g_{\mu \nu } - \Gamma ^\beta _{\ \ \rho \mu } g_{\beta \nu } - \Gamma ^\beta _{\ \ \rho \nu } g_{\mu \beta } .\\
\end{eqnarray*}

Curvature implies that parallel transport around a closed curve changes the vector being transported. Torsion corresponds to the anti-symmetric part of the connection, which refers to the asymmetry of parallel transport when exchanging the transported vector and direction of transport. Non-metricity describes the variation of the metric under parallel transport, leading to changes in the length of a vector. The Ricci scalar *R* in MAG can be expressed in terms of the Ricci scalar associated with the Levi–Civita connection as [60]


(31) \begin{eqnarray*}
R =\mathring{R}-Q+T+C+B,
\end{eqnarray*}

where the non-metricity scalar *Q*, torsion scalar *T*, mixing scalar *C* and boundary term *B* are given by


(32) \begin{eqnarray*}
Q &=& \frac{1}{4}Q^{\alpha }Q_{\alpha }- \frac{1}{2}\tilde{Q}^{\alpha }Q_{\alpha } \\
&&-\frac{1}{4}Q_{\alpha \mu \nu }Q^{ \alpha \mu \nu }+\frac{1}{2}Q_{\alpha \mu \nu }Q^{\nu \mu \alpha }, \\
T&=& -T^{\tau }T_{\tau }+\frac{1}{4}T_{\rho \mu \tau }T^{\rho \mu \tau } +\frac{1}{2}T_{\rho \mu \tau }T^{\tau \mu \rho },\\
C &=& \tilde{Q}_{\rho }T^{\rho }-Q_{\rho }T^{\rho } +Q_{\rho \mu \nu }T^{\nu \rho \mu },\\
B &=& \mathring{\nabla }_{\rho } (Q^{\rho }- \tilde{Q}^{\rho }+2T^{\rho } ),
\end{eqnarray*}

with $Q_{\alpha }=g^{\mu \nu }Q_{\alpha \mu \nu }$ and $\tilde{Q}_{\alpha }=g^{\mu \nu }Q_{\mu \alpha \nu }$ denoting the two independent traces of the non-metricity tensor, and $T^{\mu }=T^{\nu \mu }_{\nu }$ the trace of the torsion tensor. The general action of MAG may be constructed as an arbitrary function of the fundamental geometric quantities built from curvature, torsion and non-metricity. Under suitable conditions, this general framework reduces to $f(R)$ gravity [61,62], $f(T)$ gravity [60,63,64] or $f(Q)$ gravity [59,65], depending on whether the action depends solely on curvature, torsion or non-metricity, respectively. The action for these modified-gravity theories can be written uniformly as


(33) \begin{eqnarray*}
S =\int d^4 x \sqrt{-g} \,\bigg [\frac{1}{16\pi G} f(X)+\mathcal {L}_{\mathrm{m}}\bigg ],
\end{eqnarray*}

where *X* represents $R, T$ or *Q*; $R, T$ and *Q* denote the Ricci scalar, torsion scalar and non-metricity scalar, respectively; and $\mathcal {L}_{\mathrm{m}}$ is the matter Lagrangian density respectively. Under the flat FRW metric, $\mathrm{d} s^2=\mathrm{d} t^2-a(t)^2(\mathrm{d} r^2+r^2 \mathrm{d} \theta ^2+r^2 \sin ^2 \theta \mathrm{d} \phi ^2)$, with $a(t)$ the scale factor, the modified Friedmann equations can be written effectively as


(34) \begin{eqnarray*}
3 H^2 =\rho _{\mathrm{m}}+\rho _{\mathrm{de}},
\end{eqnarray*}

(35) \begin{eqnarray*}
-2 \dot{H}-3 H^2 = p_{\mathrm{m}}+p_{\mathrm{de}},
\end{eqnarray*}

where $\rho _{\mathrm{m}}$ and $p_{\mathrm{m}}$ denote the energy density and pressure of matter, and the effective energy density $\rho _{\text{de }}$ and pressure $p_{\text{de }}$ arise from the gravitational modifications.

In $f(R)$ gravity, we have


(36) \begin{eqnarray*}
\rho _{\mathrm{de}, R}&=& \frac{1}{f_R}\bigg [\frac{1}{2}(f-R f_R)-3 H \dot{R} f_{R R}\bigg ],
\end{eqnarray*}

(37) \begin{eqnarray*}
p_{\mathrm{de}, R}&=& \frac{1}{f_R}(2 H \dot{R} f_{R R}+\ddot{R} f_{R R})\\
&& +\frac{1}{f_R}\bigg [\dot{R}^2 f_{R R R}-\frac{1}{2}(f-R f_R)\bigg ],
\end{eqnarray*}

where $R=-12 H^2-6 \dot{H}$ and $f_R=\mathrm{d} f / \mathrm{d} R, f_{R R}=$  $\mathrm{d}^2 f / \mathrm{d} R^2$. Accordingly, the effective dark-energy EoS is defined as $w \equiv p_{\mathrm{de}, \mathrm{R}} / \rho _{\mathrm{de}, \mathrm{R}}$.

Among these three commonly considered modified-gravity models, $f(R)$ gravity typically yields higher-order field equations and may suffer from potential instabilities, thus requiring careful construction. By contrast, $f(T)$ and $f(Q)$ gravity involve only second-order equations.

In $f(T)$ gravity, taking the form $f(T)=T+F(T)$, the effective energy density and pressure of the additional gravitational sector, interpreted as dark energy, can be written as


(38) \begin{eqnarray*}
\rho _{\mathrm{de}, \mathrm{T}} = -\frac{1}{2} F+T F_T ,
\end{eqnarray*}

(39) \begin{eqnarray*}
p_{\mathrm{de}, \mathrm{T}} = \frac{F-T F_T+2 T^2 F_{T T}}{2+2 F_T+4 T F_{T T}},
\end{eqnarray*}

with $T=-6 H^2$. Thus, the effective EoS parameter of dark energy is $w \equiv p_{\mathrm{de}, \mathrm{T}} / \rho _{\mathrm{de}, \mathrm{T}}$.

In $f(Q)$ gravity within the coincident gauge and under the FRW metric, the background evolution is identical, with $Q=-6 H^2$. The corresponding expressions in the coincident-gauge $f(Q)$ case can be obtained from those of $f(T)$ gravity by the replacement $T \rightarrow Q$.

Meanwhile, to obtain a healthy $f(T)$ theory, the absence of instabilities is required, which imposes the condition


(40) \begin{eqnarray*}
\Omega ^2=\frac{-\frac{f}{4}-T^2f_{TT}}{f_T+2T f_{TT}} \ge 0.
\end{eqnarray*}

For example, a model with negative $\Omega ^2$ is obviously unstable under gravitational perturbations.

For specific functional forms of *f*, these modified-gravity models can produce quintom dark-energy behavior consistent with observational data [12,66].

### The effective field theory approach

The effective field theory (EFT) approach provides a powerful and unified framework for studying dynamical dark-energy models [67,68]. In this approach, the extra scalar degree of freedom arising from the spontaneous symmetry breaking of the time translation in an expanding universe appears as a Goldstone boson. Notably, the EFT framework can incorporate both single-scalar-field theories and modified-gravity theories, including $f(R)$ gravity and Horndeski theory within curvature-based EFT [68,69], as well as $f(T)$ gravity within torsion-based EFT [70]. This unified formulation enables systematic comparisons and investigations of different modified-gravity theories within a common theoretical structure.

The most general EFT action in metric-affine gravity can be written as [40]


(41) \begin{eqnarray*}
S &=& \int d^4x\sqrt{-g}\Bigg [ \frac{M_P^2}{2}\lbrace \Psi (t)\mathring{R}+d(t)T+e(t)Q\rbrace \\
&&+\, \frac{M_P^2}{2}\lbrace g(t)T^0+h(t)Q^0+j(t)\tilde{Q}^0 \rbrace \\
&&-\, \Lambda (t)-b(t)g^{00}-k(t)Q^{000} \\
&&+\,\frac{M_P^2}{2}m(t)C \Bigg ] +S_{DE}^{(2)},
\end{eqnarray*}

where $M_p^2=1/8\pi G$ is the Planck mass, and $\Psi$, $\Lambda$, *d, e, g, h, j, b, k* and *m* are functions of the time coordinate *t*. In addition, $S_{DE}^{(2)}$ contains all operators at the perturbative level.

In the curvature-based case, the EFT form can be simplified to


(42) \begin{eqnarray*}
S &=&\int \mathrm{d}^4x \sqrt{-g}\bigg [ \frac{M_p^2}{2}\Psi (t)R-\Lambda (t)-c(t)g^{00}\bigg ]\\
&& +\, S_{DE}^{(2)}.
\end{eqnarray*}

The function $\Psi$ indicates whether the scalar field is minimally coupled. Accordingly, the effective density and pressure of dark energy in the EFT frame can be defined as


(43) \begin{eqnarray*}
\rho _{de}^{\it eff}= \frac{1-\Psi }{\Psi }\rho _m-3M_p^2H\frac{\dot{\Psi }}{\Psi } +\frac{c}{\Psi }+\frac{\Lambda }{\Psi },\\
\end{eqnarray*}

(44) \begin{eqnarray*}
p_{de}^{\it eff} = \frac{1-\Psi }{\Psi }p_m+M_p^2\frac{\ddot{\Psi }}{\Psi } +2M_p^2H\frac{\dot{\Psi }}{\Psi }+\frac{c}{\Psi }-\frac{\Lambda }{\Psi }.\\
\end{eqnarray*}

Then, the effective dark-energy EoS parameter in the general EFT framework is then given by


(45) \begin{eqnarray*}
w_{de}^{\it eff} &=& \frac{(1-\Psi )p_m+M_p^2\ddot{\Psi }+2M_p^2H \dot{\Psi }+c -\Lambda }{(1-\Psi )\rho _m-3M_p^2H\dot{\Psi }+c+\Lambda } \\
&=& -1+\frac{(1-\Psi )(\rho _m+p_m)+M_p^2\ddot{\Psi }-M_p^2H\dot{\Psi }+2c}{(1-\Psi )\rho _m-3M_p^2H\dot{\Psi }+c+\Lambda }.\\
\end{eqnarray*}

The action (33) could represents the minimally coupled single-field dark-energy model when $\Psi (t)=1$. The standard quintessence scenario has the Lagrangian in the unitary gauge


(46) \begin{eqnarray*}
{-\frac{1}{2}(\partial \phi )^2-V(\phi ) \stackrel{{\mathrm{unitary}}}{\longrightarrow } -\frac{1}{2}\dot{\phi }_0^2(t)g^{00}-V(\phi _0)}.\\
\end{eqnarray*}

Comparing this with the background action in (33), we obtain


(47) \begin{eqnarray*}
\Psi (t)=1, \quad c(t)=\frac{1}{2}\dot{\phi }_0^2(t), \quad \Lambda (t)=V(\phi _0).\\
\end{eqnarray*}

In the case of $f(R)$ gravity the action can be rewritten in the unitary gauge by identifying the background value $R^{(0)}=t$, yielding


(48) \begin{eqnarray*}
f(R) \stackrel{\mathrm{unitary}}{\longrightarrow } f_R(R^{(0)})R+f(R^{(0)})-R^{(0)}f_R(R^{(0)}).\\
\end{eqnarray*}

Then we have


(49) \begin{eqnarray*}
\Psi (t) &=& f_R(R^{(0)}), \quad c(t)=0,\\
\Lambda (t)&=& - \frac{M_p^2}{2}\lbrace f(R^{(0)})-R^{(0)}f_R(R^{(0)}) \rbrace .
\end{eqnarray*}

In $f(T)$ gravity [63] the geometry is flat and metric compatible. In the unitary gauge we have


(50) \begin{eqnarray*}
f(T) \stackrel{\mathrm{unitary}}{\longrightarrow } f_T(T^{(0)})T+f(T^{(0)})-f_T(T^{(0)})T^{(0)}. \\
\end{eqnarray*}

The non-zero terms can be obtained by comparison with Equation (32):


(51) \begin{eqnarray*}
\Psi (t)&=& - f_T(T^{(0)}), \quad d(t)=2 \dot{f_T}(T^{(0)}),\\
\Lambda (t)&=& -\frac{M_p^2}{2} \big[ f(T^{(0)})-T^{(0)}f_T(T^{(0)}) \big],
\end{eqnarray*}

Following analogous steps, the $f(Q)$ action in the unitary gauge takes the form


(52) \begin{eqnarray*}
f(Q)\stackrel{\mathrm{unitary}}{\longrightarrow } f_Q(Q^{(0)})Q+f(Q^{(0)})-f_Q(Q^{(0)})Q^{(0)}.\\
\end{eqnarray*}

The corresponding non-zero terms are


(53) \begin{eqnarray*}
\Psi (t)&=& f_Q(Q^{(0)}), \quad j(t)=-h(t)=\dot{f_Q}(Q^{(0)}),\\
\Lambda (t) &=& -\frac{M_p^2}{2} \big[f(Q^{(0)})-Q^{(0)}f_Q(Q^{(0)}) \big].
\end{eqnarray*}

In particular, the background evolution of $f(Q)$ cosmology in the coincident gauge is identical to that of $f(T)$ gravity.

### Interacting dark energy

Along the line of the quintom scenario with two fields, systems involving a scalar field coupled to neutrinos [71] or to dark matter [72,73], as well as interacting two-fluid models [74] have been considered.

The interacting dark-energy model is also an important candidate for explaining the DESI observations. It describes the existence of interactions between dark energy and dark matter, leading to energy transfer between them. As a result, an additional interaction term appears in their conservation equations, commonly denoted by $Q_{\mathrm{int}}$:


(54) \begin{eqnarray*}
\dot{\rho }_{\mathrm{de}}+3 H(1+w) \rho _{\mathrm{de}}=Q_{\mathrm{int}},
\end{eqnarray*}

(55) \begin{eqnarray*}
\dot{\rho }_{\mathrm{dm}}+3 H \rho _{\mathrm{dm}}=-Q_{\mathrm{int}},
\end{eqnarray*}

The interaction term $Q_{\mathrm{int}}$ is often parameterized as a function of the Hubble constant, the dark-matter energy density and the dark-energy density, for example $\xi H\rho _{\mathrm{dm}}$, $\xi H\rho _{\mathrm{de}}$ and other possible forms [75]. Interacting dark-energy scenarios can also be derived from a Lagrangian perspective. By introducing a coupling between a scalar-field dark-energy component $\phi$ and a spinor dark-matter field $\psi$, a corresponding interaction term $Q_{\mathrm{int}}(\phi ,\psi )$ can also be obtained [76].

Owing to the presence of the interaction, this contribution can be interpreted as modifying the effective equation-of-state parameter of dark energy, thereby altering the properties of the dark sector. Of course, its impact on cosmic evolution also depends on the specific form of the interaction. Suitable interaction forms can render the effective dark-energy equation of state consistent with the quintom-like behavior indicated by the DESI result [8,77].

### Compare different models

The quintom behavior is a feature based on observations. It indicates that the effective dynamical dark-energy component extending beyond the $\Lambda$CDM framework must exhibit an EoS parameter that crosses $-1$. Moreover, quintom characteristics suggest that dark energy is unlikely to be a simple cosmological constant or a canonical single scalar field; rather, it may possess more complex evolutionary dynamics.

To circumvent the conditions imposed by no-go theorem and realise the quintom evolution, various models have introduced different approaches. In single-scalar-field dark-energy frameworks, multi-scalar models introduce additional fields as extra degrees of freedom. However, achieving a crossing of $w=-1$ typically requires a phantom field with a negative kinetic term, thereby violating the NEC. This kind of ghost field suffers from quantum instabilities [78].

Single-scalar-field models with higher-derivative terms introduce coupling between the kinetic term and higher-derivative operators. The presence of such couplings allows the scalar field to exhibit richer evolutionary behavior, thereby enabling quintom realizations. Nevertheless, generic higher-derivative models still contain ghost modes. To obtain a stable theory, requirements must be imposed on the couplings, as realised in degenerate higher-derivative models [79].

Modified-gravity theories address the problem from a different perspective by extending the gravitational action of general relativity. The additional gravitational terms can be interpreted as an effective dynamic dark-energy component that drives the accelerated expansion of the Universe. Since the effective equation-of-state parameter is an effective representation of the gravitational terms not directly related to the physical energy density, $w_{eff}$ can naturally cross the $-1$ boundary without physically violating the NEC. Modified-gravity theories typically introduce additional degrees of freedom, which also modify the evolution of perturbations. The specific forms of modified-gravity models also need to satisfy the corresponding stability conditions, and the behavior of the extra degrees of freedom remains an active area of research [59,60].

Interacting dark-energy models generate dynamical behavior for dark energy by incorporating a coupling between dark energy and dark matter. This affects not only the dynamics of dark energy but also the evolution of dark matter. The effective quintom behavior also depends sensitively on the form of the interaction term. It is therefore necessary to constrain the form or parameter space of the interaction term to ensure that its impact on large-scale structure aligns with observations and maintains a stable interactive system [8,75].

There are also approaches that explore the realization of the quintom scenario using fermionic fields [80] and holographic dark-energy models [81,82]. Some studies have interpreted the DESI result from the perspective of dark matter, for example by allowing a non-zero equation-of-state parameter for dark matter [83], along with many other mechanisms discussed in the literature.

As discussed above, a quintom-like evolution of $w(z)$ can arise from a wide range of microphysical theoretical frameworks, which is highly degenerate at the background level. It is difficult to distinguish among these models using only observations at the level of cosmological background evolution. To break this degeneracy and identify the most plausible theoretical explanation for quintom dark energy, it is essential to incorporate the behavior of perturbations. Employing effective field theory, we can investigate the perturbation-level properties characteristic of various model categories.

Modified-gravity models influence not only the evolution of the cosmic background but also structure formation, gravitational waves, gravitational potentials and other related phenomena. Meanwhile, interacting dark-energy models affect physical processes associated with dark matter. Models involving interactions with photons will also produce effects on CMB polarization, which will be discussed in detail in the following section. Future multi-messenger observations at the perturbation level— including measurements of structure growth, gravitational lensing and slip, gravitational waves and CMB polarization—will provide tests and constraints on dynamical dark-energy scenarios, offering deeper insights into the fundamental nature of quintom dark energy.

## IMPLICATIONS OF QUINTOM COSMOLOGY

### Interactions of quintom dark energy with ordinary matter

As a dynamical field, quintom dark energy is expected to couple directly to ordinary matter in the Universe. In general, one imposes a shift symmetry, $\phi \rightarrow \phi +c$, on such interactions. The shift symmetry requires that the dark-energy scalar couple to matter fields only through derivative interactions. At the leading order, the derivative couplings take the form $\partial _{\mu }\phi J^{\mu }$, which generate spin-dependent forces. Such forces between microscopic particles cannot superpose into long-range forces between unpolarized macroscopic objects. In addition, the shift symmetry suppresses large radiation corrections.

Couplings to the baryon current, $\partial _{\mu }\phi J_B^{\mu }$, or to the *B*-*L* current, $\partial _{\mu }\phi J_{B\mathrm{-}L}^{\mu }$, can be used to construct the baryogenesis or letpogenesis models [84,85], in which the observed baryon asymmetry is generated in thermal equilibrium in the early Universe. During the evolution of quintom dark energy, $\dot{\phi }$ does not vanish; these coupling terms therefore violate the Lorentz and CPT symmetries. This mechanism explains how baryon asymmetry can be produced thermally in such models.

The dynamical dark-energy field may also couple derivatively to photons through a Chern–Simons term,


(56) \begin{eqnarray*}
\mathcal {L}_{CS}=\frac{c}{M}\partial _{\mu }\phi A_{\nu }\tilde{F}^{\mu \nu },
\end{eqnarray*}

where *c* is the coupling constant, *M* is a mass scale from the perspective of effective field theory and $\tilde{F}^{\mu \nu }=(1/2)\epsilon ^{\mu \nu \rho \sigma }F_{\rho \sigma }$ is the dual electromagnetic field tensor. Through this Chern–Simons coupling, the evolution of the quintom field in the Universe induces CPT violation in the photon sector, which may be potentially observed by CMB polarization experiments. For a single light ray, the polarization direction of the photon rotates under the Chern–Simons coupling as the photon propagates from the source to the observer. This rotation can be expressed in terms of the Stokes parameters as


(57) \begin{eqnarray*}
(Q\pm iU)^{\prime }=\exp (\pm i2\chi )(Q\pm iU),
\end{eqnarray*}

and the rotation angle depends on the field difference [86]


(58) \begin{eqnarray*}
\chi =\frac{c}{M}\Delta \phi \equiv \frac{c}{M}[\phi (x_s)-\phi (x_o)].
\end{eqnarray*}

For CMB photons, the source corresponds to the last-scattering surface, $x_s=x_{lss}$. This in turn modifies the CMB power spectra and, in particular, generates non-vanishing $TB$ and $EB$ correlations [87], where $T, E$ and *B* denote the temperature, E-mode polarization and B-mode polarization, respectively. By assuming an isotropic rotation angle $\chi =\bar{\chi }$, one obtains the full set of equations for the rotated CMB spectra [88]:


(59) \begin{eqnarray*}
{C^{\prime }}_l^{TE}&=& C_l^{TE}\cos 2 \bar{\chi },\\
{C^{\prime }}_l^{TB}&=& C_l^{TE}\sin 2 \bar{\chi },\\
{C^{\prime }}_l^{EE}&=& C_l^{EE}\cos ^2 2 \bar{\chi }+C_l^{BB}\sin ^2 2 \bar{\chi },\\
{C^{\prime }}_l^{BB}&=& C_l^{EE}\sin ^2 2 \bar{\chi }+C_l^{BB}\cos ^2 2 \bar{\chi },\\
{C^{\prime }}_l^{EB}&=& \frac{1}{2} \left(C_l^{EE}-C_l^{BB} \right)\sin 4\bar{\chi }.
\end{eqnarray*}

The first detection of a CMB rotation angle was reported in [88], where a non-zero rotation angle $\bar{\chi }=-6.0\pm 4.0\, {\rm deg}$ was mildly favored by CMB polarization data from the three-year WMAP observations and the January 2003 Antarctic flight of BOOMERanG.

Owing to the dynamical nature of quintom dark energy, the rotation angle need not be strictly isotropic. The quintom field exhibits both temporal evolution and spatial fluctuations. Its inhomogeneous distribution on the last-scattering surface induces anisotropies in the rotation angle [86], such that $\chi =\bar{\chi }+\delta \chi$. From $\chi =({c}/{M})[\phi (x_s)-\phi (x_o)]$, the anisotropic rotation angle is given by $\delta \chi =({c}/{M})\delta \phi (x_{lss})$, which depends on the perturbation of the quintom field at the last-scattering surface. This leads to additional distortions of the CMB spectra and may be detectable in future CMB experiments [86].

### Quintom cosmology in the very early Universe

In standard cosmological frameworks such as the $\Lambda$CDM model and conventional inflationary scenarios, the evolution of the early Universe inevitably traces back to an initial singularity—an extreme state of divergent curvature, energy density and temperature at which general relativity ceases to be valid. To resolve this fundamental issue, alternative models have been proposed, among which bounce cosmology offers a promising route to non-singular early-Universe evolution [61,89,90]. In this section, we demonstrate that non-singular models can emerge naturally within the framework of quintom cosmology.

Bounce cosmology encompasses scenarios in which the Universe initially undergoes a phase of contraction, reaches a finite minimum scale (the bounce) and subsequently enters an expanding phase. Near the bounce, the momentary violation of the NEC becomes essential. To achieve a smooth transition from the contracting to the expanding phase and eventually recover the hot Big Bang conditions, the EoS parameter must evolve from $w<-1$ to $w>-1$.

This behavior can be understood by examining the dynamics of the scale factor $a(t)$ and the Hubble parameter $H(t)$. During contraction, $\dot{a} < 0$, whereas during expansion, $\dot{a} > 0$. At the bounce point, $\dot{a} = 0$, and a successful bounce requires $\ddot{a} > 0$ in its neighborhood. Equivalently, the Hubble parameter transitions from $H < 0$ to $H > 0$, passing through $H = 0$ at the bounce, which implies that $w<-1$ in its neighborhood. However, to avoid a subsequent Big Rip—as occurs in purely phantom dark-energy models—the Universe must evolve toward a standard thermal history, necessitating a transition from $w < -1$ to $w > -1$. This quintom-like evolution is therefore not only compatible with bounce cosmology but also a requirement for its viability. Bouncing solutions can also be obtained in quintom models through phenomenological realizations [90]. Beyond phenomenological constructions, bounce cosmology can also be implemented within fundamental field-theoretic models. Models of this class have been studied in [90], and the dynamics of their perturbations have been analyzed in [91].

Moreover, within the same theoretical framework, it is possible to construct cyclic cosmologies, in which the Universe experiences a turn-around phase during expansion that triggers contraction and subsequently generates the next bounce, resulting in an oscillatory cyclic evolution. The idea of a cyclic Universe has since been revisited in various contexts, including higher-dimensional string theory [92] and loop quantum cosmology [93,94]. An appealing aspect of cyclic quintom cosmology is its ability to avoid both Big Rip and Big Crunch singularities while naturally incorporating periodic phases of acceleration. Furthermore, since the scale factor grows from cycle to cycle, the model predicts a progressively flatter universe, thereby addressing the flatness problem without fine-tuning. One such cyclic model, based on a parameterized quintom EoS, has also been proposed to alleviate the coincidence problem [95].

The emergent-Universe scenario posits a past-eternal cosmos with a finite, non-zero scale factor as $t\rightarrow -\infty$, thus avoiding the Big Bang singularity. In its original formulation, the Universe is asymptotically Einstein static before entering an inflationary phase [96]. Quintom fields allow such an emergent phase even in a flat FRW model [97]. In these cases, the analytic solutions demonstrate a smooth transition from a quasi-static initial phase to radiation-like expansion, without encountering a curvature singularity.

In addition, many other non-singular models of the very early Universe have been proposed within nonconventional theoretical frameworks. For instance, adding higher-derivative ‘ghost condensate’ terms to the action allows a smooth bounce in ekpyrotic models [98]. It has been shown that fermion condensation of the Nambu–Jona–Lasinio type can also induce a non-singular bouncing solution [99]. In beyond-Horndeski scalar-tensor theories, one can design a non-singular bounce as well [100].

## SUMMARY AND FUTURE PROSPECT

In this paper, we have briefly reviewed quintom models of dark energy. We summarised the historical development of dark energy, from the static cosmological-constant paradigm to the observationally supported dynamical behavior. When the dark-energy EoS $w(z)$ deviates from $-1$, its phenomenology can be broadly categorized into quintessence, phantom and quintom. The latest DESI DR2 results, combined with complementary cosmological datasets, provide substantive evidence for dynamic dark energy. Both the CPL parametrization and non-parametric approaches consistently indicate that the dark-energy EoS parameter $w(z)$ evolves across $-1$, corresponding to a quintom-B scenario.

We have shown that, owing to the no-go theorem, quintom models require additional degrees of freedom. Multi-field scalar theories, higher-derivative single-field frameworks, modified gravity and interacting dark energy can realize the quintom scenario and serve as candidate theories for dark energy in light of current observational trends. An effective field-theory framework can bridge the scalar-field and geometric approaches, providing a powerful and unified method for studying different dynamical dark-energy models.

We have examined the interactions of the quintom field with ordinary matter and pointed out that such interactions lead to spin-dependent forces and generate the matter–anti-matter asymmetry in the Universe. Quintom interactions with photons induce a rotation of CMB polarization. This effect can serve as an additional test of quintom dark-energy models, complementary to measurements of the EoS.

Forthcoming DESI data releases, with improved statistical accumulation and extended redshift coverage, are expected to yield a deeper understanding of the fundamental nature and dynamical evolution of dark energy. Euclid and Rubin are expected to provide cross-checks of DESI results through complementary BAO measurements. Future supernova surveys, such as ZTF and the Rubin Observatory, will provide important constraints on the low-redshift Universe. Meanwhile, next-generation CMB experiments, including the Simons Observatory, CMB-S4 and AliCPT, will refine our understanding of the early Universe and tighten constraints on dynamical dark-energy models. Notably, precise measurements of the CMB TB and EB power spectra from future CMB polarization observations could probe a possible Chern–Simons coupling between dark energy and photons, offering a novel test for dynamical dark-energy scenarios.
